# Violence risk assessment as a medical intervention: ethical tensions

**DOI:** 10.1192/pb.bp.113.043315

**Published:** 2014-04

**Authors:** Ashimesh Roychowdhury, Gwen Adshead

**Affiliations:** 1 St Andrew’s Healthcare, Northampton; 2 Broadmoor Hospital, Crowthorne, Berkshire

## Abstract

Risk assessment differs from other medical interventions in that the welfare of the patient is not the immediate object of the intervention. However, improving the risk assessment process may reduce the chance of risk assessment itself being unjust. We explore the ethical arguments in relation to risk assessment as a medical intervention, drawing analogies, where applicable, with ethical arguments raised by general medical investigations. The article concludes by supporting the structured professional judgement approach as a method of risk assessment that is most consistent with the respect for principles of medical ethics. Recommendations are made for the future direction of risk assessment indicated by ethical theory.

The assessment of risk of violence by mental health professionals has been the subject of ethical criticism because of the potential harm done to patients without justification in terms of patient benefits or respect. However, it is also a core duty of psychiatric services mandated by a number of clinical and legal frameworks.

The assessment of risk of violence is a core function of all psychiatric services, particularly in forensic psychiatry.^1^ As such, it can, and should, be considered in a similar light to other types of medical assessment and intervention. This includes not only the statistical properties of the process itself, such as sensitivity and specificity (further defined below) but also using accepted models of biomedical ethics for its analysis. In this special article we argue that if violence risk assessment is a clinical intervention (like an X-ray or magnetic resonance imaging scan), then it should be subject to the same bioethical framework as other medical interventions. In support of this argument, we explore four related questions: first, why clinicians become involved in violence risk assessment at all; second, whether traditional bioethical frameworks help with an analysis of the ethical issues. The third question looks at the key features of different approaches to risk assessment and whether they pose common or unique ethical dilemmas; and the fourth question explores whether traditional bioethics assists us to know what approach to the risk assessment process clinicians ought to adopt, both now and into the future. To address these questions, we have used analogous arguments about the assessment of risk in general medicine, for example in cardiac care.

## Why are clinicians involved in violence risk assessment?

The *NHS Outcomes Framework*^2^ states that an overarching objective for the National Health Service (NHS) is to reduce avoidable harm. In relation to mental health services, this includes reducing violence by people with mental disorders to themselves or others. Although mental disorders make a relatively small contribution to the risk of violence to others compared with risk factors such as substance misuse,^3^ there is thought to be sufficient contribution to generate a *prima facie* duty to reduce the risk. For example, when either suicides or homicides by mentally disordered patients occur, there is usually an internal inquiry, a possible externally commissioned inquiry and mandatory information-sharing with the National Confidential Inquiry into Suicide and Homicide^4^ to explore ‘lessons learnt’. The costs of homicides and suicides by people with mental illness are therefore considerable, not just in terms of the emotional harm caused to victims and their families, as well as perpetrators themselves, but also in terms of financial costs to healthcare providers.

The General Medical Council (GMC) guidance on good medical practice^5^ and confidentiality^6^ sets out the duties and responsibilities of a doctor. Although the GMC states that ‘the care of [my] patient is [my] first concern’,^5^ it recognises a role for doctors in the welfare of others, especially children or those at risk of infectious disease. For example, if an HIV-positive person refuses to disclose their status to an intimate partner who may be at risk of contracting HIV, the GMC supports a doctor disclosing information about risk to the partner, despite the affected person’s flat refusal. The doctor must weigh the harms that are likely to arise from non-disclosure of information against the possible harm of disclosure both to the patient and to the overall trust between doctors and patients. The onus is on the clinician to assess the risks of the situation in order to make a decision compatible with this guidance.

The NHS staff guidance on confidentiality supports a much broader remit for disclosure and states that clinicians are justified in disclosing clinical information which leads to the detection, prevention and investigation of serious crime.^7^ The Royal College of Psychiatrists offers similar advice on confidentiality and the duty to disclose,^8^ as does the Department of Health in its advice on risk assessment.^1^

In legal terms, Article 8.1 of the Human Rights Act 1998 supports the right to privacy and family life, but permits restrictions if, Any interference or restriction on privacy would have to be proportionate to the identified risk to self and others, which entails an onus to assess those risks. The Public Health (Control of Disease) Act 1984 restricts the liberty of individuals who are thought to be carriers of communicable diseases. Further, mental health legislation in England and Wales gives power to approved professionals to detain citizens on the basis of perceived risk to self or others,^9^ which not only implies that risk assessment is part of every implementation of the Mental Health Act 1983, but also requires that the assessment needs to be justified and performed to a good standard.

‘necessary in a democratic society for the interests of national security, public safety or the economic well being of the country, the prevention of disorder or crime, for the protection of health and morals, or for the protection of the rights and freedoms of others’.

There is case law, both from the UK and the USA, which supports the duty of healthcare professionals to protect the public. A particularly famous case is that of Tarasoff^10^ where the Californian Supreme Court found that healthcare professionals (including therapists) had a duty to protect the public. In the case of Egdell,^11^ the court found in favour of a doctor who sent an unfavourable report about a patient to the Home Office. The patient sued for negligence on the basis of a breach of confidentiality, but the court found that there was a duty to share information about danger with public bodies. In the case of Palmer,^12^ the court found that a health trust had no duty of care to unnamed and unidentifiable victims of a man whose mental condition made him a risk to others, although one inference might be that if there was an identifiable victim, healthcare professionals would have duty of care to them, as well as to their patient.

Both the Bradley review^13^ and the impact of the Department of Health’s publication that followed the review, *Improving Health, Supporting Justice*^14^ (applicable to England and Wales) imply that mental health services play a key role in public protection. An example is the use of psychiatric evidence at sentencing and parole hearings for prisoners detained under Indeterminate Sentences for Public Protection (IPPs). These sentences were introduced by the Criminal Justice Act 2003 (applicable across the UK) for individuals who had committed a ‘specified offence’ (of a seriously violent or sexual nature) and who were thought to pose a real risk of ‘dangerousness’ to others if released. Courts and parole boards ask for psychiatric evidence as to future risk, which affects the detention of the prisoner. Although IPPs are now no longer open to judges as a sentencing option, existing orders still run and psychiatrists may be asked to assess risk in offenders on IPPs.

Indefinite detention on the grounds of risk is also possible under the Mental Health Act for individuals with a mental disorder. Individuals convicted of a crime that would warrant imprisonment may be detained in psychiatric settings under Section 37 of the Act (a hospital order) and the detention may be ‘without limit of time’ if a Section 41 (restriction order) is also imposed. The Section 41 order can only be imposed by a crown court judge who has heard from psychiatrists who have assessed the defendant as posing a risk of serious harm to others. Unlike IPPs, there is no minimum tariff and the offence does not have to meet the IPPs ‘dangerousness’ level. This has led some to argue that the Section 37/41 disposal unfairly discriminates against those with a mental disorder, who can be detained indefinitely and usually for much longer than if they had received a prison sentence.^15^

## Traditional bioethical frameworks

Beauchamp & Childress^16^ suggested a ‘four principles’ approach to ethical decision-making in general medicine. This framework argues that doctors have at least *prima facie* duties to respect the autonomous choices of individuals; act in ways that promote their welfare and minimise harm; and respect commonly held principles of justice. The framework includes two schools of moral philosophy: deontology, which examines the intentions behind an action to determine its rightness, and consequentialism, which looks at the outcomes of any action to determine its moral rightness. The principles of respect for autonomy and justice reflect a deontological stance, whereas the principles of beneficence and non-maleficence reflect a consequentialist stance. It has been commented that the relative dominance of these two schools is itself a consequence of the dominance of left-brain thinking^17^ in Western culture, where the left hemisphere logically calculates risks and benefits and finds reasons for what people should or should not do.

Although the four principles framework was widely accepted as a basis for general medical bioethics, other frameworks have also been influential. For example, virtue ethics^18^ emphasises the character of the clinician and the value of lived experience (this could be seen as more reflective of right-hemisphere thinking) and Fulford’s values-based practice framework^19^ addresses the process of balancing facts and values in bioethics.

These schools of ethical thought have been explored and developed in relation to general psychiatry, especially values-based practice and virtue ethics. English mental health legislation reflects similar ethical values in the presumption of capacity that underlies the Mental Capacity Act 2005 and the emphasis on risk and benefit that justifies the overriding of autonomy in the English Mental Health Act.

However, there has been much less analysis of how ethical frameworks apply to forensic psychiatry, especially in relation to the duty to respect justice processes. Beauchamp & Childress’ analysis of respect for justice^16^ addresses distributive justice only in relation to resource allocation; it makes no mention of how doctors contribute to judicial processes and how they should avoid acting unjustly towards people who are detained or imprisoned. Forensic psychiatrists may look for ethical guidance from the GMC and the Royal College of Psychiatrists, but these bodies do not provide guidance on how psychiatrists should conduct risk assessments that are ethically sound, i.e. that promote the welfare of patients, do no harm and respect the principles of justice - not just distributive justice but justice as fairness, honesty and objectivity.^20^

Classical ethical theory has particular limitations in relation to forensic psychiatry and risk assessment. Deontological theory struggles when ethical duties seem to conflict, such as the duty to protect the public *v*. the duty of care to the individual service user. The theory does not allow for the ranking of duties, leaving practitioners without a practical solution. There is also no rule-based way to rank order the values of each outcome for each stakeholder in a risk assessment process to come up with the best course of action. Consequentialist theory raises complex questions about how to assess likely benefits, and empirical questions about the accurate estimation of probabilities of certain outcomes occurring. In the case of violence risk assessment, there are concerns that there is a lack of accurate information (in relation to possible negative outcomes) that would be needed to justify intrusions into liberty and autonomy. It might even be argued that there is no actual information about possible risk but only anxiety of others that risky events may occur.

In ethical terms, risk assessments cause tension between the welfare of the individual and the welfare of others. Both the restriction of liberty and the breach of privacy are usually justified with reference to the benefit of harm prevented. Patients assessed as high risk are likely to lose their liberty (which is a harm); but if the process of risk assessment is flawed, then they are also treated with less justice than other people (which is a wrong). If misleading or false data are used as a justification for detention, then this is an unjust process, just as it is unjust to admit false or misleading evidence into a criminal trial. There is a parallel argument here with research ethics: if the techniques used in the research are flawed or the methodology is incoherent, then it cannot be ethically justifiable to put humans through procedures that might do them harm, because there can be no meaningful benefit that will justify the harm done.^21^

## Methodology of risk assessment: common and unique ethical issues

Risk is defined as the probability that a harmful event will occur. It is a negative form of chance; we do not, for example, talk about the ‘risks’ of having good weather. Risk assessment is therefore about assessing the likelihood that something bad will happen, which will cause distress and harm to others: or, to put it another way, risk = hazard + outrage (www.psandman.com).

Risk is a multidimensional construct, its most common dimensions being its nature, its probability, its severity, its imminence and its frequency. Risk assessment must address all of these areas to enable a risk management plan to be formed, and must also include the possibility of beneficial or positive things happening that reduce the negative effects or outcomes. The output of any risk assessment usually leads to a decision to take action that is intended to reduce either the likelihood of the negative event happening or the negative impact of the event.

Risk assessment usually leads to one of three conclusions:

1. patients assessed for the first time are assessed as low, medium or high risk
2. patients being reassessed are deemed to be the same risk as in the previous assessment
3. patients being reassessed are deemed higher or lower risk than last time.

Risk assessment may also include an assessment of mental health/illness and a treatment review, since a key theoretical feature of risk assessment is the relationship between some types of psychiatric symptoms and risk of violence.

Specifically in relation to psychiatry, if an individual with a mental disorder is assessed as being at high risk of causing severe harm in the near future, steps will be taken to reduce the likelihood of his acting harmfully, and also to reduce the impact of any harm. Legal powers to detain that patient and restrict his actions may be used as a risk management strategy. It is also possible to reduce risk by (a) warning the potential victim, (b) altering the mental state that gives rise to the risk, and (c) both.

There are three recognised approaches to risk assessment:^22^

- unstructured clinical judgement,
- actuarial risk assessment tools, and
- structured professional judgement.

### Unstructured clinical judgement

Unstructured clinical judgement refers to a purely clinical opinion on risk, without necessarily following a set structure. ‘Unstructured’ can be a misleading term: there may be structure imposed on the evaluation but it will vary from clinician to clinician and perhaps even from one patient to another assessed by the same clinician. This approach has the advantage of being flexible, quick and idiographic (person centred), and it was the traditional way of assessing risk for many years.

### Actuarial risk assessment tools

Actuarial approaches to assessing risk are similar to those operated by the insurance industry. These approaches are based on established statistical relationships between measurable predictor and outcome variables. The outcome of the assessment is determined by fixed and explicit rules, and there is no attempt to elucidate an explanatory model between the predictor variable and the outcome variable: the only important issue is the strength of the statistical correlation.

Actuarial tools, such as the Violence Risk Appraisal Guide (VRAG),^23^ sometimes combine static predictors (that do not require clinical judgement to rate) with dynamic factors that still require clinical judgement. The process remains actuarial in that the total score is used to reflect the risk and gives rise to probabilistic statements. This enhances reliability and statistical predictive validity over unstructured judgement.

### Structured professional judgement

The structured professional judgement (SPJ) approach involves clinical judgement with a structured application. There has been a proliferation of SPJ tools for different risks, such as the Historical, Clinical and Risk Management Scales (HCR-20) for violence,^24^ the Risk for Sexual Violence Protocol (RSVP) for sexual offending,^25^ and the Short Term Assessment of Risk and Treatability (START) for multiple risk domains.^26^

Each SPJ instrument follows a core methodological process. First, there is collation of comprehensive background information, followed by a rating of items on the tool for their absence, partial presence or presence (usually on a 0-2 scale or ‘yes’, ‘no’ or ‘maybe’ rating). The items are derived from the empirical literature for their association with the outcome variable, but, unlike actuarial tools, they are not optimised from one sample, which enhances generalisability. Each item is operationally defined to enhance interrater reliability. Most SPJ tools consider both historical/static risk items and dynamic risk items. The relevance of each item to future risk can be rated. Case-specific items can be considered: these are items not part of the core tool, but which the clinician feels are highly relevant to the risk behaviour being evaluated.

Once items are rated, the next step is to construct a risk formulation (an explanatory/causative model) as to how the risk items combine to produce the outcome. This is often expressed in a risk scenario or risk specificity statement that addresses questions such as: What risk needs to be considered? Who is at risk and over what time period? How likely is that risk and how severe may it be? Unlike actuarial tools, multiple scenarios can be considered, depending on the decision in question. The risk is then summarised as low, medium or high, which then guides development of a risk management plan.

Before describing their unique ethical issues, there are three areas where any type of risk assessment method can come under ethical criticism.

### Consent

If risk assessment is a medical intervention which is being applied to a patient, with potentially harmful ‘side-effects’, consent is usually obtained from the patient. Failure to obtain consent is a basis for action in negligence (as a breach of duty of care, under tort law, the law that governs civil wrongs between one individual and another).

An analogous situation is the use of the exercise tolerance test (ETT) to assess cardiac risk. Before an ETT, the patient undergoes a consent procedure that should enable them to make an informed decision about participation. This would include relevant information about the nature and purpose of the procedure, the risks involved, the consequences of having or not having the test, likely outcomes and next steps were the test to be ‘positive’ or ‘negative’, and how and with whom those results would be shared. This would be evidenced by a written consent form. In the case of a patient who lacked capacity, a best interest decision would have to be made about the risks and benefits of the procedure. A patient who fails an ETT cannot be detained in hospital even if this is in his own best interests, unless he lacks capacity.

If consent is obtained for an ETT (which carries some degree of risk to health), then one may ask why consent should not be obtained in the case of violence risk assessment. The positive determination of moderate or increased risk may lead to harm; namely detention and/or significant restrictions on liberty, and disclosure of personal information. The patient could be provided with information about the risks and benefits of risk assessment, and refusal to participate could be interpreted in the light of other known data. Without such a consent process, the patient might argue that an intervention done without his consent and which results in his continued detention is a tortious act, especially if it is done badly or using a tool known to be flawed. Even if the law allows breaches of confidentiality for legal purposes (e.g. preparing a risk assessment for a criminal proceeding), explicit consent could still be sought, or at least the defendant could be advised that he does not have to participate.

### Distributive justice and resources

Emphasis on risk and risk assessment in mental health may also breach the principle of justice (fairness) in terms of resources allocation. In the past 10 years, there has been a diversion of resources towards forensic mental health: a fifth of the entire mental health budget is spent on 4000 forensic beds.^27^ Since 2002, forensic spending has risen by 140%, compared with 42% for the rest of the mental health service. Spending on healthcare has risen most in those perceived as highest risk, but this does not necessarily correlate with highest need. Assessment of cost-effectiveness of this has not been undertaken.^28^

### Low positive predictive validity for individuals leading to over-restriction

Szmukler^29^ and others have suggested that the base rates of violence (i.e. the known prevalence of a specified type of violent behaviour within a given population over a period of time) in those with mental disorder are so low that even a highly accurate risk assessment instrument would result in significant errors, particularly low positive predictive validity, meaning the number of people predicted to be violent who then are violent.

Table 1 shows the potential outcomes of a violence risk assessment. Patients assessed as being at high risk of violence may actually be violent (’true positive’ outcome) or may desist from future violent behaviour (’false positive’). Similarly, patients assessed as being at low risk of future violence may actually be violent (’false negative’) or may desist (’true negative’). Clearly, the aim of accurate risk assessment is to maximise the frequency of true positive and true negative predictions.

**Table 1:** Contingency table showing potential outcomes of risk assessment for violence

	Outcome at follow-up	
Prediction	Violent	Non-violent
High risk	True positive (TP)	False positive (FP)
Low risk	False negative (FN)	True negative (TN)

Sensitivity: TP/TP+FN, specificity: TN/TN+FP, positive predictive value: TP/TP+FP, accuracy: TP+TN/TP+FP+TN+FN.

To illustrate, example figures are shown in Table 2 for a sample of 1000 service users where the base rate of violence is 10% and the tool has a sensitivity and specificity of 90%. Using these numbers, the risk assessment appears to be highly accurate in that 90% of patients are allocated to true positive and true negative conditions. However, the positive predictive value (PPV), which is the proportion of those identified by the risk assessment instrument as high risk and who actually are violent is only 50%, which is the level of chance prediction, or, in other words, only as accurate as tossing a coin.

This argument cast doubt on whether any form of violence risk assessment could be carried out with sufficient accuracy. This situation posed significant ethical challenges in the management of individual patients because it was difficult to confidently justify any restrictions as being proportionate to the risk. More recently, this pessimism has been countered by studies showing that the base rates of violence in those with mental disorder are higher than first thought, as well as by advances in statistical methods.

**Fig 1:**
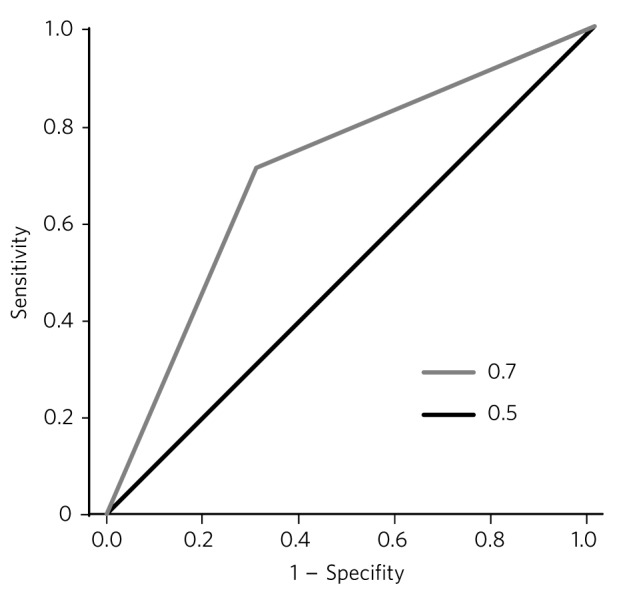
Example receiver operating characteristic (ROC) curve.

Technically, risk assessment instruments do predict better than chance. Their accuracy is currently based on receiver operating characteristics (ROC) and the area under the curve (AUC) which provide an index of precision.^30^ The ROC is a plot of the true positive rate on the *y*-axis (the test sensitivity) against the false positive rate on the *x*-axis (the test specificity) (Fig. 1). The AUC represents the likelihood of correct risk prediction with the chance level being 0.5 which is represented by a straight line, where for every true positive identified there is a false positive. For different cut-off points on the risk assessment instrument, the true positive and false positive rates can be plotted with the resulting curve representing the ‘optimal fit’ that gives the greatest AUC. An AUC of 1.0 gives perfect prediction and an AUC of 0.7 or above represents a large effect size. An AUC of 0.75 means that if someone was violent, there is a 75% chance that the risk assessment instrument would have identified him as being at higher risk for violence. One meta-analysis^31^ showed that most risk assessment tools of the actuarial and SPJ variety show AUCs of 0.7-0.8.

Unlike the PPV, the ROC method is unaffected by base rates. However, the base rate of violence in risk assessment remains important because of the difficulty of translating the AUC value into clinically meaningful information by itself.^32^ To elaborate, if a risk assessment instrument was used as a screening test, and those identified as likely to be violent remained in secure care, then, for any given period, the number of patients we require to be detained (number needed to detain, NND) in order to prevent one violent act can be calculated. Numbers needed to detain is the inverse of PPV, and, like the ROC, derives from sensitivity, specificity and the base rate. For example, Buchanan,^32^ using data from the VRAG (sensitivity 0.73, specificity 0.63) found that with a violence base rate of 10%, the VRAG would require the detention of 5 people to prevent 1 unwanted act, whereas chance prediction (AUC = 0.5) would require detention of 10. The NND rises as the base rate of violence falls.

**Table 2:** Contingency table showing potential outcomes of risk assessment for violence for 1000 service users where base rate of violence is 10%

	Violent, *n*=100	Non-violent, *n*=900
High risk, *n*	90	90
Low risk, *n*	10	810

On the face of it, the NND figure of 5 compares very favourably with an analogous measure of clinical effectiveness, the number needed to treat (NNT). A possible result of a ‘positive’ ETT is that the person would be recommended for a coronary artery bypass graft and the NNT to prevent 1 death over a 5-year period is 53.^33^ However, it could be argued that preventing a death from a cardiac event and preventing a death by violence are not comparable harms - one is natural (albeit pathological), whereas violence is neither natural nor evidence of pathology.

Perhaps a fairer comparison would be the NND to prevent one act of fatal harm to others, not all acts of violence. In this instance, the NND would rise considerably from 5, due to the low prevalence of fatal harm. Therefore, base rates, if known, provide a context in which to make proportionate decisions resulting from a risk assessment. A key challenge in psychiatry is that base rates are often not known, are low and vary for different types of violence.

The issue is not unique to mental health. For example, the negative predictive value (NPV) of an exercise electrocardiography (ECG) for future cardiac events is high, meaning those predicted not to have a cardiac event will probably not have one;^34^ however, the PPV is only 13.5%.^34^ Despite these inaccuracies, the exercise ECG is a routine part of cardiac care and an extra cost to the NHS. The key ethical difference is that those deemed to be at high risk of a cardiac event are not detained in hospital to prevent that event or improve their cardiac health. Citizens are left alone to deal with their own risk as they see fit. Citizens with histories of mental illness, however, are not left to make their own decisions, but may be detained using statutory legislation. The question is then why we persist in attempts to predict risk behaviour, given the error rates? Even though the chance of preventing one serious event is low, it could be argued that the benefit outweighs the harm done by detention based on false positives. Walker^35^ suggested that, given the problems with the predictive accuracy of risk assessments, this argument may only be ethically tenable for preventing future harm of a type that the person has already committed, as opposed to future harm that is hypothetical and different in nature or severity.

In his book, *Treating Violence*, Maden noted that we assign different values to a false positive and to a false negative when it comes to considering risk.^22^ Said differently, as a culture, professionally and societally, a false negative outcome (i.e. failing to identify and intervene to prevent a high-risk behaviour) is much less acceptable and is to be avoided, as compared with a false positive. This value differential may be strengthened in clinical practice by two factors: first, that false negatives are known and visible, and second, that healthcare professions suffer the effects of false negatives (the corresponding serious investigations and hostile scrutiny that can follow false negative events); however, it is patients who suffer the effects of risk management based on a false positive premise.

Another premise, and to elaborate an earlier point, is that the belief that human behaviour is predictable through logical analysis of a set of variables is itself a reflection of left-brain thinking, to the detriment of right-brain thinking that may recognise the detrimental effect this assumption may have on issues such as trust between patients and psychiatrists.

A recent study^36^ confirmed that, although the NPV of risk assessment tools was high (i.e. those predicted to be non-violent tended to be non-violent), the PPV was only 0.41 on average. The chance of being detained unnecessarily is therefore high and making decisions about detention or release based purely on risk assessment prediction cannot be said to be ‘evidence based’. The study argues that decisions need to be made on the basis of a ‘wider assessment’ which would include contextual and clinical factors.

We will now turn to a more detailed review of the three approaches to risk assessment, highlighting the point that an understanding of ethical issues does favour the SPJ approach over other methods, despite the fact that it is not necessarily more reliable or valid.

Clinical judgement alone has significant drawbacks.^37^ It has been criticised for being subjective and impressionistic, lacking transparency, reliability and validity, and leading to idiosyncratic decisions based on the experience of the assessor. There are heuristic biases in decision-making by clinicians, who (like other professionals) tend to overlook evidence that does not fit with their pre-existing theories about the data.^38^ It can be seen as profession-centric, lacking patient involvement, and thus breaching the principle of autonomy. Lack of validity and reliability can be construed as harmful, given the potential consequences of the assessment.

Although actuarial methods removed some of the idiosyncrasies from clinical risk assessment and improved reliability, there have also been concerns that these methods are insufficiently clinical in nature, leading to inaccuracy and perverse outcomes. For example, Logan^39^ noted that purely actuarial approaches to violence risk assessment indicated that those who killed a female had lower reoffending rates than those who killed a male, hence it could be seen as a protective factor for future violence: a finding that seemed counterintuitive and lacking in validity.

Actuarial tools lack utility in helping clinicians make the range of decisions required in secure care. They can only answer the question: ‘In a group with these characteristics, how likely is it that a member of the group will commit the outcome in question within the specified time period?’. They cannot answer the question of whether this person will commit another violent act, one that may be different from the first. Issues that exercise clinicians such as severity or imminence of risk, in different situations, are not helped by actuarial scores. Returning to the discussion about base rates, actuarial tools cannot help the clinician differentiate between different types of violence, only some of which may be seen as the duty of services to try to prevent (e.g. because it appears linked to mental disorder).

Actuarial risk assessment is based on static factors that cannot change, which is anomalous in therapeutic settings where positive change is the aim of therapeutic interventions. Actuarial tools also exclude the use of an explanatory model to connect risk factors and risk assessment, which means that there is little that either the patient or the clinician can do to understand how to reduce risk. Similarly, actuarial tools do not consider moderating factors (variables that affect the strength and direction of the relationship between the predictor and criterion variable), mediating factors (intervening factors between the independent and dependent variable) and protective factors (that reduce the likelihood of the outcome variable).^40^ The exclusion of such evidence is *prima facie* evidence of an unjust process, and one lacking in potential beneficence.

Structured professional judgement focuses only on risk areas most relevant to the person, and does not invite discussion of any imaginable risk factor: which means that irrelevant discussion of risk is excluded from the process, which is more just. By the same argument, in the assessment it is possible to include protective and moderating factors that reduce risk, which would be more consistent with a fair process of assessment. Tools, such as the START, incorporate a strengths scale on all items as well as a vulnerability scale. Studies have found that both scales are independently predictive of risk behaviour, and that if the strengths score is the same or higher than the vulnerability score, the likelihood of risk behaviour in a 90-day follow-up period is much reduced.^41^

Operational definitions of risk factors and how these relate to the risk behaviour allow scrutiny and challenge to the risk assessment, upholding autonomy and participation principles. Structured professional judgement tools incorporate both static and dynamic variables, the latter suggesting targets for treatment and change, thus leading to the (possibility) of patient benefit. Still, SPJ is not without its ethical concerns. Since these assessments are often done as part of a group discussion, group dynamics may lead to an unfair process in which a dissenting voice (often one which argues for reduced risk) is shouted down.^42^ The forensic patient’s voice is unlikely to be given the same weight as professional voices and may often be assumed to be unreliable and untrustworthy.

### What does ethical theory suggest about how we ought to conduct risk assessment?

The SPJ approach has evolved as the gold-standard approach recommended by the Department of Health, at least in part because its clinical content gives it more obvious validity and makes it more like the ‘wider assessment’ (compared with other approaches) that is needed to justify loss of liberty. By emphasising prevention rather than prediction, it parallels other medical interventions that seek to prevent disasters and therefore makes a more traditional therapeutic alliance more possible. It is of note that this relies on the tool being used properly: SPJ tools used as checklists of risk factors without construction of risk scenarios or a risk management plan remains harmful and unethical practice.

In conclusion, the recent evolution of best practice in risk assessment has addressed (knowingly or unknowingly) some of its key ethical concerns. At present, the SPJ approach to risk assessment should be used and supported within psychiatric services as being most sensitive to ethical concerns. Its methodology allows for patient involvement and challenge, upholding the principle of autonomy. Consideration of dynamic risk factors, strengths and protective factors allows the possibility of patient benefit. Emphasis on strengths as well as risk is to be welcomed as respectful of the patient’s dignity and empirically more sound. Harm done by over-prediction may be reduced by consideration of all the dimensions of risk, risk scenario construction and forming of a proportionate management plan. However, further developments are needed to make risk assessment more ethically aware. A more ethically nuanced approach to risk assessment might first seek consent, then take into account the patient’s voice and narrative of their risk, for example, including patients in scenario generation. Further research may identify ‘static’ strengths that relate to violence that are not merely the absence of risk factors, for instance positive attachment to an adult role model; and may provide more information about the correlates of nature, imminence and severity of risk than just probability. This can help clinicians make more informed decisions, for both patient and wider societal benefit.
